# From a Sanatorium

**Published:** 1918-03-16

**Authors:** John Ferguson


### From a Sanatorium.

Not ours by day on shell-scarred fields to fight,
Nor ours o' nights the snary seas to scour,
Ours but to drowse from hour to languorous hour
Throughout the sluggish day and slow-paced night;
Pampered and stuffed, we rest ignobly here,
While, drenched in shrapnel spray and hail of steel,
With engin'ry of death the continents reel,
And tremble with the clang of martial gear.

We are as men that dream; and, soldier-wise,
We man feigned trenches here in sleepish ease,
And stand in dreams amid the shrapnel spray;
And when night falls, on some conceived emprise,
We board our men-o'-war and sail away,
And with imagined searchlights sweep the seas.

John Ferguson.

